# Supplementary material for: A Novel Demulsifier with Strong Hydrogen Bonding for Effective Breaking of Water-in-Heavy Oil Emulsions
Source: Int J Mol Sci. 2023 Sep 30;24(19):14805. doi: 10.3390/ijms241914805 (PMC10573199; doi:10.3390/ijms241914805)
Supplement: Supplementary file 1 [file ijms-24-14805-s001.zip › ijms-2594839-supplementary.pdf]

International Journal of
Molecular Sciences

Article

A Novel Demulsifier with Strong Hydrogen Bonding for Effective Breaking of Water-in-Heavy Oil Emulsions

Xiao Xia [1,2,†], Jun Ma [1,2,*,†], Fei Liu [1,2], Haifeng Cong [3,4] and Xingang Li [3,4]

[1] Department of Chemical Engineering, School of Chemistry and Chemical Engineering, Guizhou University, Guiyang 550025, China; xiaxiao2021@163.com (X.X.); ce.feiliu@gzu.edu.cn (F.L.)
[2] Guizhou Key Laboratory for Green Chemical and Clean Energy Technology, Guiyang 550025, China
[3] School of Chemical Engineering and Technology, Tianjin University, Tianjin 300072, China; conghaifeng@tju.edu.cn (H.C.); lxg@tju.edu.cn (X.L.)
[4] Zhejiang Institute of Tianjin University, Ningbo 315201, China
* Correspondence: jma3@gzu.edu.cn
† These authors contributed equally to this work.

Supporting information

1. Molecular weight analysis of demulsifiers

Fig. S1 *shows the gel permeation chromatography of different demulsifiers, transverse axis is the logarithmic value of molecular weight, vertical axis is* the weight-normalized area of the slice (dwt/d(logM)) and cumulative percentage of the area (Ht%). The absolute value of the area of the slice is divided by the slope of the calibration curve at the adjusted elution volume of the slice. The ratios of area to slope for all the slices are summed, and then each ratio is divided by the sum.

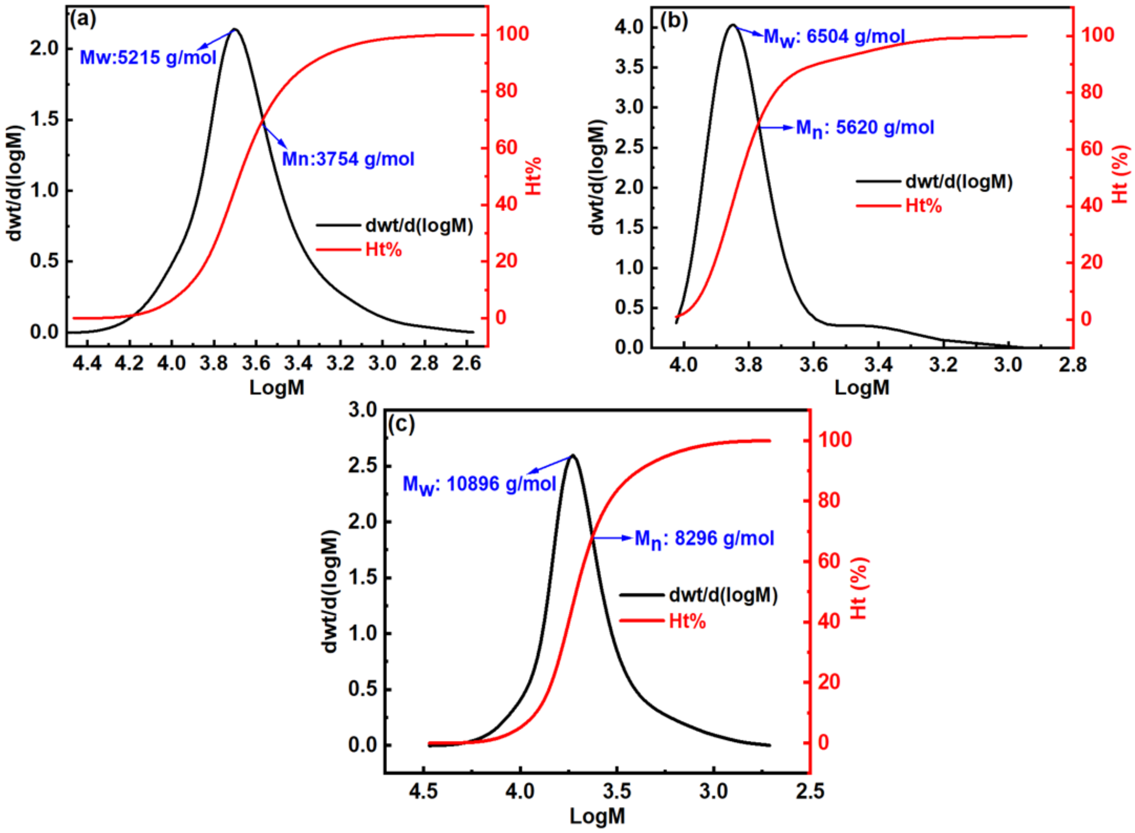

Figure S1. Gel permeation chromatogram of different demulsifiers (a: FAP, b: JXGZ, c: h-PAMAM).

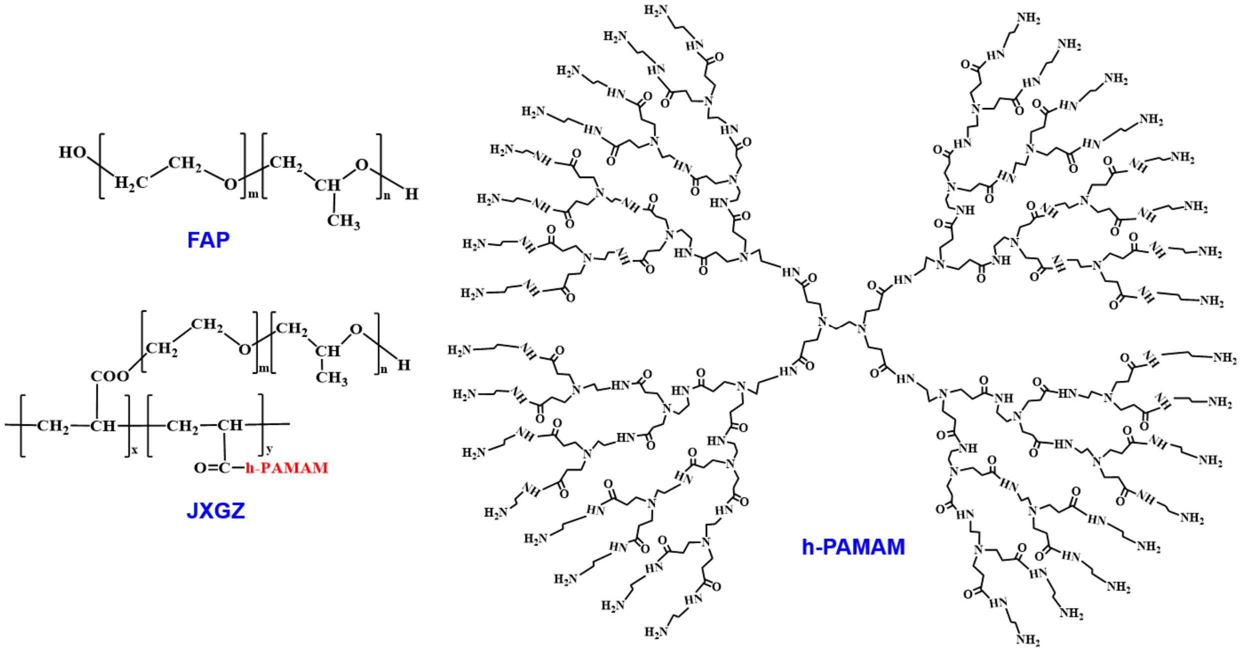

Figure S2. The structure diagram of FAP, JXGZ and h-PAMAM demulsifiers.

2. Synthetic demulsifier

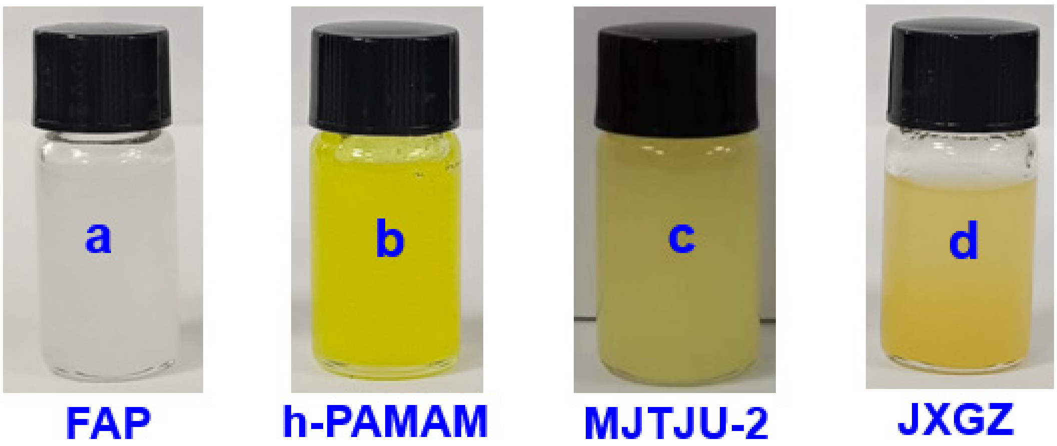

Figure S3. The photograph of different demulsifiers.

3. Optimization of molecular structure model and molecular dynamics calculations

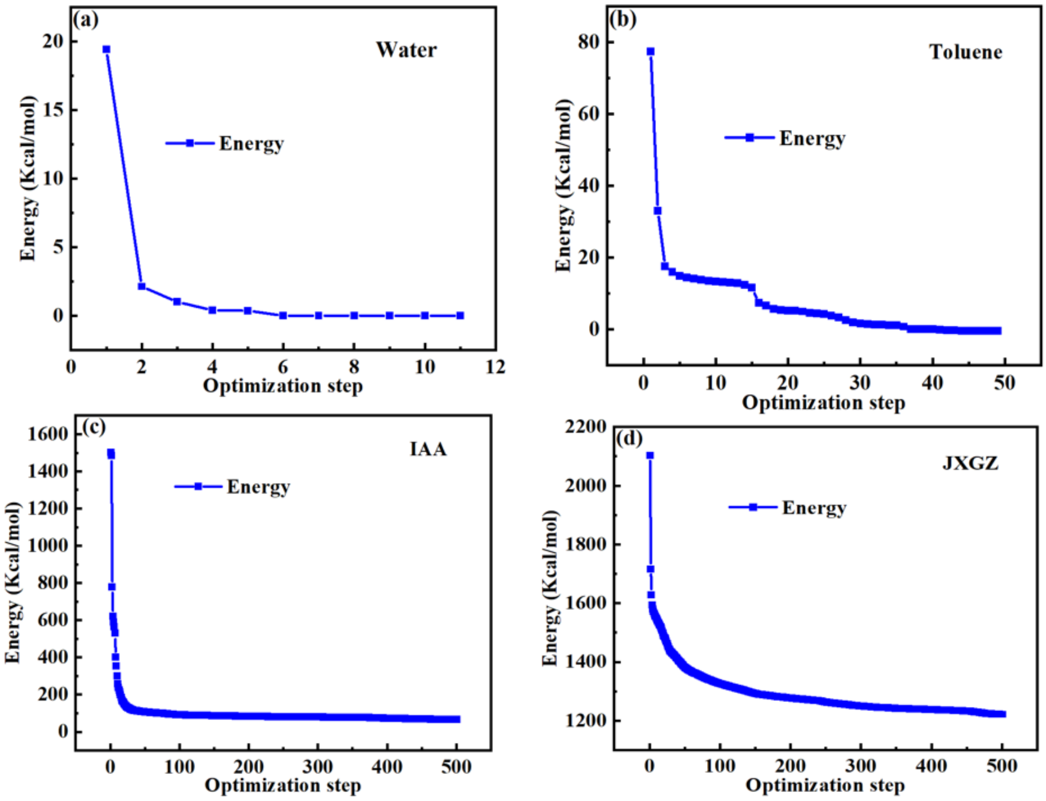

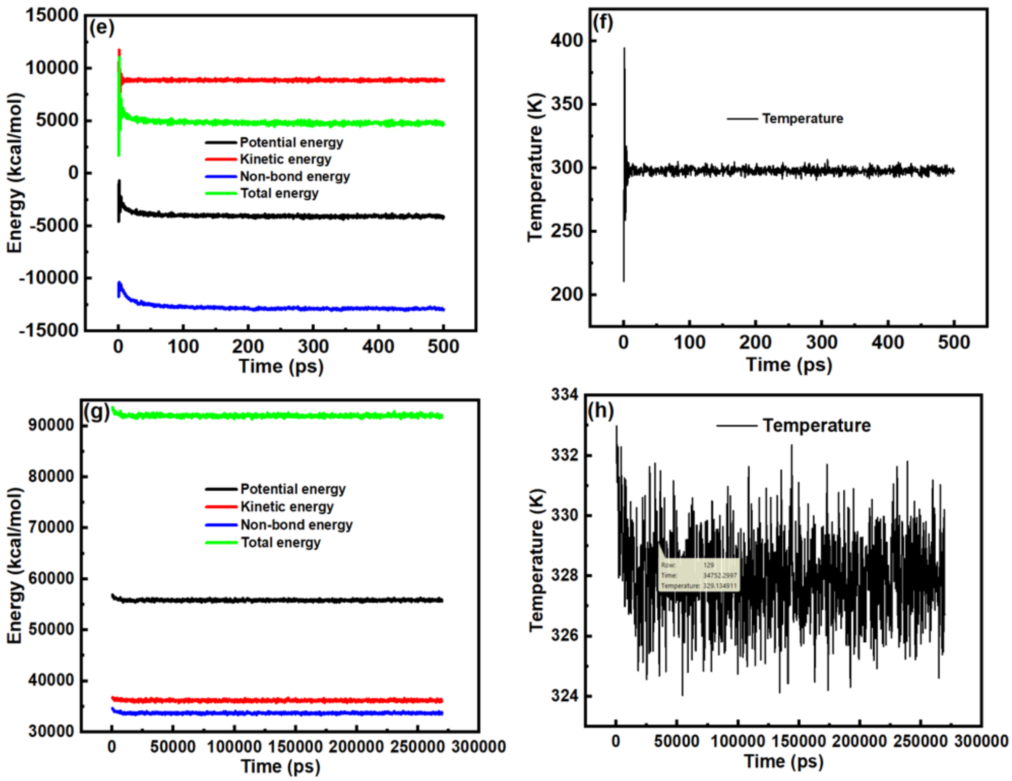

Figure S4. The change curve of energy with simulation steps of optimized molecular structures (a-d), and the variable curve of energy and temperature with simulation time in all atom molecular dynamics simulation (e and f) and dissipative particle dynamics simulation (g and h).

4. Dissipative particle dynamics (DPD) simulation theories

The DPD theory is described from the following three aspects: the force of DPD simulation, the algorithm of DPD simulation, and the calculation process of DPD simulation.

4.1. The Force in DPD Simulation

Hoogerbrugge and Koelman developed DPD simulation in the present form of Espanol[1, 2]. In the DPD simulation, the elementary atoms or molecules are defined as a bead, and the "bead" is used to replace some groups or substances in molecules. The motion behavior of beads conforms to Newton's equation of motion, as shown in formulas (1) and (2) [3].

$$\frac{dr_i}{dt} = v_i \qquad (1)$$

$$m_i \frac{dv_i}{dt} = f_i \quad (2)$$

Where r_i, v_i, and f_i represent the displacement, velocity, and total acting force of the i th bead, respectively.

In the DPD system, in order to calculate conveniently, the mass of each bead in the system is divided into the same, and the non dimensional unit mass is used to represent the mass of each bead ($m_i = 1$). According to formula (2), the total force of each bead can be obtained is equal to its acceleration in numerical value. The total acting force of each bead can be divided into three parts: the conservative force F_{ij}^C (the force is only related to the initial and final positions of the beads, indicating the repulsion between the beads), the dissipative force F_{ij}^D (the force is determined by the relative velocity between beads) and the random force F_{ij}^R (the force is used to describe the collision between beads). The total acting force of each bead in the system can be described by equation (3) [4].

$$f_i = \sum_{j \neq i}(F_{ij}^C + F_{ij}^D + F_{ij}^R) \quad (3)$$

Where i and j of the subscript indicate the interaction between different beads. The physical meaning of the summation symbol is that the forces exerted on other beads are summed without considering the beads i within the specific truncation radius r_c. According to the position coordinates of beads in the system, the magnitude and direction of three forces of beads will be obtained. The interaction between beads can be determined by truncation radius r_c (in DPD simulation system, the truncation radius is dimensionless unit length, $r_c = 1$). If the distance between beads in the system is larger than the truncation radius, there is no interaction between beads.

The conservative force is calculated according to formula (4). Where a_{ij} is the repulsion force parameter between beads. The maximum repulsion force between beads is shown in equation (5). Groot and Warren [1,

5] compared the free energy of molecular structure model in DPD system with Flory Huggins theory of polymer system [6-9], and obtained the repulsive force parameter between different beads a_{ij}, similar beads a_{ii}. They found that the Flory Huggins parameter x_{ij} satisfy the relationship shown in equation (6). The density of the system is set to 3 so that the fluid in the system possess the compressibility similar to water.

$$F_{ij}^{C} = \begin{cases} a_{ij}(1 - |r_{ij}|)\hat{r_{ij}} & r_{ij} < r_c \\ 0 & r_{ij} \geq r_c \end{cases} \quad (4)$$

$$r_{ij} = r_j - r_i,\ \hat{r_{ij}} = r_{ij}/|r_{ij}| \quad (5)$$

$$\alpha_{ij} = \alpha_{ii} + \frac{x_{ij}(T)}{0.306} = \alpha_{ii} + 3.27x_{ij}(T) \qquad \rho = 3 \quad (6)$$

The dissipative force is described by equation (7). The dissipation force is proportional to the relative velocity between the two beads. Its role is to reduce the relative vector between the beads, where $\omega^D(r_{ij})$ is a short-range weight function. The dissipative force can maintain the momentum conservation between the beads, ensuring the momentum of the whole system is conserved.

$$F_{ij}^{D} = \begin{cases} -\gamma\omega^D(r_{ij})(\hat{r_{ij}} \cdot v_{ij})\hat{r_{ij}} & r_{ij} < r_c \\ 0 & r_{ij} \geq r_c \end{cases} \quad (7)$$

The random force is described by equation (8), where θ_{ij}（t） is a random variable of compound Gaussian distribution and possesses the following relationship: $< \theta_{ij}(t) >= 0$ and $< \theta_{ij}(t)\theta_{kl}(t') >= (\delta_{ik}\delta_{jl} + \delta_{il}\delta_{jk})\delta(t - t')$. In the simulation system, the random force is not added to each moving bead separately, but as pairs of beads. Therefore, the total linear momentum can be conserved.

$$F_{ij}^{R} = \begin{cases} \sigma\omega^R(r_{ij})\theta_{ij}\hat{r_{ij}} & r_{ij} < r_c \\ 0 & r_{ij} \geq r_c \end{cases} \quad (8)$$

4.2. The Algorithm of DPD Simulation

In DPD simulation, the improved Verlert velocity algorithm [10] is usually used to solve Newton's equation of motion, the solution process is as follows: (1) The position coordinate, velocity and direction of the bead at

the next moment are determined by the current position coordinate, speed and direction of the bead and the force applied; (2) the force of the bead at the next moment is calculated according to the current position coordinate, speed and direction, and then the speed is corrected to complete a cycle. The formulas used in the process are given as follows from equations (9) to (12) .

$$r_i(t+\delta t) = r_i(t) + \delta t v_i(t) + \frac{1}{2}(\delta t)^2 f_i(t) \quad (9)$$

$$\widetilde{v}_i(t+\delta t) = v_i(t) + \lambda \delta t f_i(t) \quad (10)$$

$$f_i(t+\delta t) = f_i[r(t+\delta t), \tilde{v}(t+\delta t)] \quad (11)$$

$$v_i(t+\delta t) = v_i(t) + \frac{1}{2}\delta t[f_i(t) + f_i(t+\delta t)] \quad (12)$$

4.3. The Calculation Process of DPD Simulation

The general calculation process of DPD is shown in **Fig. S5.** The main steps are as follows:

(1) Setting the initial conditions, including the total number of beads in the system, system temperature, system density, operation steps and time.

(2) Newton's equation of motion was solved by computer.

(3) The average temperature, potential energy and total energy of the system were calculated.

In the process of DPD simulation, the most important thing is to accurately obtain the repulsion force between beads, which plays the decisive role in the accuracy of the final dynamic calculation results. After the calculation and analysis of the forces all beads in the system, the Material Studio software will solve Newton's equation of beads to get the position coordinates, speed and direction of beads at a specific time. It will further calculate the temperature, energy and pressure of the system to keep the system at constant temperature and constant pressure, and finally through the cycle until the initial set calculation time is completed.

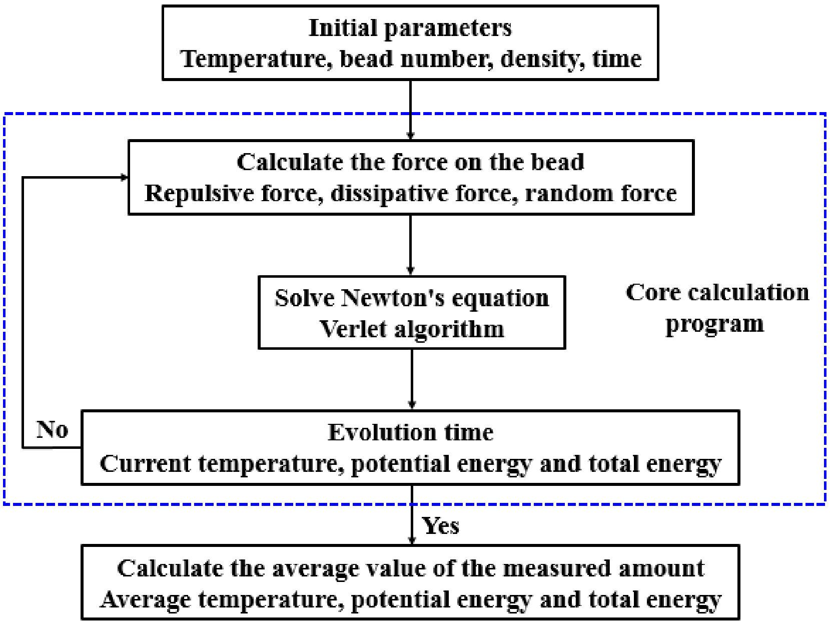

Figure S5. DPD calculation process.

Table S1. The atomic clusters represented by each bead and repulsive force parameters between the beads.

Automic clusters	Beads	A0	A1	A2	A3	A4	A5	S1	S2	S3	S4	S5	S6	S7	S8	S9	P1	P2	P3	P4	P5	P6	P7	P8	P9	P10	P11	P12	P13	P14	P15	T1	T2	W
$C_{14}H_{14}NOS$	A0	25	82	35	31	69	26	57	39	60	44	59	36	57	58	60	30	41	41	38	31	37	40	87	48	72	36	53	28	77	30	33	83	69
$C_{15}H_{14}S$	A1	82	25	56	50	40	96	82	73	80	51	79	70	75	72	80	63	76	82	86	74	73	87	104	86	93	92	80	72	101	73	70	103	118
$C_{14}H_{13}OS_2$	A2	35	56	25	39	51	43	82	61	81	57	81	69	75	76	82	58	66	68	37	61	66	68	69	31	91	60	76	58	97	58	48	101	88
$C_{14}H_{13}OS_2$	A3	31	50	39	25	53	29	76	57	82	62	82	58	76	78	82	57	72	70	36	62	65	70	78	39	91	69	79	58	97	58	50	102	98
$C_{15}H_{14}S$	A4	69	40	51	53	25	74	76	72	81	56	82	79	78	76	81	67	82	86	94	78	77	87	109	89	97	83	86	83	103	78	70	104	123
$C_{14}H_{14}NOS$	A5	26	96	43	29	74	25	56	39	62	43	61	49	65	56	61	33	39	40	48	32	43	41	87	36	69	35	55	29	76	30	35	80	79
C_6H_{14}	S1	57	82	82	76	76	56	25	30	26	30	26	30	26	25	25	34	31	39	56	36	30	38	95	50	27	67	29	63	27	63	25	28	48
$C_5H_{12}O$	S2	39	73	61	57	72	39	30	25	30	35	30	26	30	30	30	26	27	25	38	26	28	27	46	36	30	36	26	33	32	33	30	33	37
C_5H_{12}	S3	60	80	81	82	81	62	26	30	25	32	25	29	25	25	25	34	31	39	54	36	30	38	95	50	26	67	29	64	26	63	25	27	47
$C_{16}H_{32}$	S4	44	51	57	62	56	43	30	35	32	25	32	35	30	30	32	36	35	45	58	40	34	43	98	57	38	70	38	64	39	63	28	42	60
C_5H_{12}	S5	59	79	81	82	82	61	26	30	25	32	25	29	25	25	25	34	31	39	58	37	31	38	96	51	26	67	29	64	26	64	26	27	47
$C_5H_{12}O$	S6	36	70	69	58	79	49	30	26	29	35	29	25	30	30	29	27	25	28	38	26	26	28	49	35	30	36	27	36	31	35	27	33	36
C_6H_{14}	S7	57	75	75	76	78	65	26	30	25	30	25	30	25	25	25	34	30	40	57	36	31	39	95	50	27	67	30	64	27	63	25	28	48
C_6H_{14}	S8	58	72	76	78	76	56	25	30	25	30	25	30	25	25	25	34	31	40	54	36	30	39	94	51	27	67	30	63	28	62	25	29	48
C_5H_{12}	S9	60	80	82	82	81	61	25	30	25	32	25	29	25	25	25	35	31	39	57	37	30	38	94	50	26	67	29	64	26	64	26	27	47
$C_6H_{10}O_3$	P1	30	63	58	57	67	33	34	26	34	36	34	27	34	34	35	25	26	26	35	26	26	26	40	32	36	36	28	35	38	34	26	40	48
$C_5H_8O_2$	P2	41	76	66	72	82	39	31	27	31	35	31	25	30	31	31	26	25	29	33	29	26	29	41	30	31	41	25	37	33	36	26	34	46
$C_4H_6O_2$	P3	41	82	68	70	86	40	39	25	39	45	39	28	40	40	39	26	29	25	37	25	29	25	33	36	39	31	29	31	42	32	31	42	54
$C_3H_4O_2$	P4	38	86	37	36	94	48	56	38	54	58	58	38	57	54	57	35	33	37	25	35	38	40	46	27	54	40	33	35	60	42	37	58	37
$C_6H_{10}O_2$	P5	31	74	61	62	78	32	36	26	36	40	37	26	36	36	37	26	29	25	35	25	28	26	31	34	37	29	28	30	39	29	29	41	53
$C_5H_8O_2$	P6	37	73	66	65	77	43	30	28	30	34	31	26	31	30	30	26	26	29	38	28	25	29	43	29	31	41	25	38	33	37	26	34	46
$C_4H_6O_2$	P7	40	87	68	70	87	41	38	27	38	43	38	28	39	39	38	26	29	25	40	26	29	25	42	38	39	34	29	36	41	38	30	42	53
$C_3H_4O_2$	P8	87	104	69	78	109	87	95	46	95	98	96	49	95	94	94	40	41	33	46	31	43	42	25	48	93	38	37	46	98	43	57	99	75
$C_3H_4O_2$	P9	48	86	31	39	89	36	50	36	50	57	51	35	50	51	50	32	30	36	27	34	29	38	48	25	50	35	29	33	55	30	31	55	42
C_2H_4	P10	72	93	91	91	97	69	27	30	26	38	26	30	27	27	26	36	31	39	54	37	31	39	93	50	25	66	28	64	25	63	28	25	45
C_3H_7NO	P11	36	92	60	69	83	35	67	36	67	70	67	36	67	67	67	36	41	31	40	29	41	34	38	35	66	25	39	27	70	26	36	71	66
C_2H_7N	P12	53	80	76	79	86	55	29	26	29	38	29	27	30	30	29	28	25	29	33	28	25	29	37	29	28	39	25	38	29	40	26	30	40
$C_3H_8N_2O$	P13	28	72	58	58	83	29	63	33	64	64	64	36	64	63	64	35	37	31	35	30	38	36	46	33	64	27	38	25	68	27	33	71	62
C_2H_6	P14	77	101	97	97	103	76	27	32	26	39	26	31	27	28	26	38	33	42	60	39	33	41	98	55	25	70	29	68	25	67	28	25	46
$C_3H_8N_2O$	P15	30	73	58	58	78	30	63	33	63	63	64	35	63	62	64	34	36	32	42	29	37	38	43	30	63	26	40	27	67	25	31	70	63
C_6H_6	T1	33	70	48	50	70	35	25	30	25	28	26	27	25	25	26	26	26	31	37	29	26	30	57	31	28	36	26	33	28	31	25	30	45
CH_4	T2	83	103	101	102	104	80	28	33	27	42	27	33	28	29	27	40	34	42	58	41	34	42	99	55	25	71	30	71	25	70	30	25	47
H_6O_3	W	69	118	88	98	123	79	48	37	47	60	47	36	48	48	47	48	46	54	37	53	46	53	75	42	45	66	40	62	46	63	45	47	25

References

1. Groot, R.; Warren, P. Dissipative particle dynamics: Bridging the gap between atomistic and mesoscopic simulation. *J. Chem. Phys.* **1997**, *107*, 4423-4435.
2. Aphinyan, S.; Geethalakshmi, K.; Yeo, J.; Shakouri, A.; Ng, T. Numerical characterization of ultraviolet ink fluid agglomeration and the surfactant effect in nanoinkjet printing. *Polym. Adv. Technol.* **2017**, *28*, 1057–1064.
3. Du, C.; Ji, Y.; Xue, J.; Hou, T.; Tang, J.; Lee, S.T.; Li, Y. Morphology and performance of polymer solar cell characterized by DPD simulation and graph theory. *Sci. Rep.* **2015**, *5*, 16854.
4. Pan, G.; Manke, C. Developments toward simulationS of entangled polymer melts by dissipative particle dynamics (DPD). *Int. J. Mod. Phys. B* **2003**, *17*, 231–235.
5. Espanol, P.; Warren, P. Statistical mechanics of dissipative particle dynamics. *Europhys. Lett.* **1995**, *30*, 191.
6. Fredrickson, G.; Liu, A.; Bates, F. Entropic corrections to the Flory-Huggins Theory of polymer blends: Architectural and conformational effects. *Macromolecules* **1994**, *27*, 2503–2511.
7. Bawendi, M.; Freed, K. Systematic corrections to Flory–Huggins theory: Polymer–solvent–void systems and binary blend–void systems. *J. Chem. Phys.* **1988**, *88*, 2741–2756.
8. Maiti, A.; Wescott, J.; Kung, P. Nanotube–polymer composites: Insights from Flory–Huggins theory and mesoscale simulations. *Mol. Simul.* **2005**, *31*, 143–149.
9. Zhao, Y.; Inbar, P.; Chokshi, H.; Malick, A.; Choi, D. Prediction of the thermal phase diagram of amorphous solid dispersions by Flory–Huggins Theory. *J. Pharm. Sci.* **2011**, *100*, 3196–3207.
10. Laurent, A. Review of algorithms used in molecular dynamics simulations. *Undergrad. Res. Nat. Clin. Sci. Technol. (URNCST) J.* **2019**, *3*, 1–11.
